# Increased risk of congenital heart disease in twins in the North of England between 1998 and 2010

**DOI:** 10.1136/heartjnl-2015-307826

**Published:** 2015-09-28

**Authors:** K E Best, J Rankin

**Affiliations:** 1 Institute of Health & Society, Newcastle University, Newcastle upon Tyne, UK; 2 PHE: Regional Maternity Survey Office, Newcastle upon Tyne, UK

## Abstract

**Objective:**

To examine the relative risk (RR) of congenital heart disease (CHD) in twins compared with singletons, according to chorionicity.

**Methods:**

Twins and singletons with CHD notified to the Northern Congenital Abnormality Survey between 1998 and 2010 were included in this population-based study. Information on chorionicity was obtained from the Northern Survey of Twins and Multiple Pregnancy. Prevalence was calculated as the number of cases occurring in live births, late miscarriages (20–23 weeks), stillbirths (≥24 weeks) and terminations of pregnancy for fetal anomaly, per 10 000 total births. The risk of CHD in twins compared with singletons was estimated using Poisson regression.

**Results:**

There were 399 414 singleton births of which 2984 (0.7%) had CHD. Among 11 871 twin births, 154 (1.3%) had CHD; one twin was affected by CHD in 2.5% of twin pregnancies. Of 8605 dichorionic (DC) births and 2317 monochorionic (MC) births, 96 (1.1%) and 47 (2.0%) were associated with CHD. Compared with singletons, twins were at significantly increased risk of CHD (RR=1.73, 95% CI 1.48 to 2.04; p<0.001). MC twins were at 82% significantly increased risk of CHD compared with DC twins (RR=1.82, 95% CI 1.29 to 2.57; p<0.001). The RR of severe and mild CHD was particularly high in MC twins compared with singletons (292% increased risk, RR=3.92, 95% CI 1.25 to 12.30, p=0.02 and 207% increased risk, RR=3.07, 95% CI 2.20 to 4.28; p<0.001).

**Conclusions:**

Compared with singletons, twins were at increased risk of CHD, the risk being substantially higher among MC twins. This information is important for health professionals when counselling women with twin pregnancies.

## Introduction

There is an increased risk of congenital anomalies in multiple compared with singleton pregnancies.^1–4^ The risk among twins that share a placenta, monochorionic (MC) twins, exceeds that of twins that do not share a placenta, dichorionic (DC) twins.^1^ The risk of congenital heart disease (CHD) among twins is less well researched. While several case series have investigated the prevalence of CHD in twins,^5–8^ few studies have compared the rate with singletons.^1^ ^4^ ^9^ Of those that have, the risk of CHD was significantly increased by between 47% and 63% in twins.^1^ ^4^ ^9^ Even fewer studies have examined the risk of CHD by chorionicity. In Glinianaia *et al*'s^1^ study, there was a 30% and 50% increased risk of CHD in MC and DC twins compared with singletons, but this only reached significance in DC twins. Herskind *et al* examined the relative risk (RR) in twins compared with singletons according to zygosity, a proxy for chorionicity given that all dizygotic twins are DC and approximately two-thirds of monozygotic twins are MC. Herskind *et al*^9^ reported significantly increased risks of 35% and 30% in monozygotic and dizygotic twins, respectively.

The aim of this study was to examine the RR of CHD in twins compared with singletons, according to chorionicity and CHD severity.

## Methods

### Data sources

**Figure 1:**
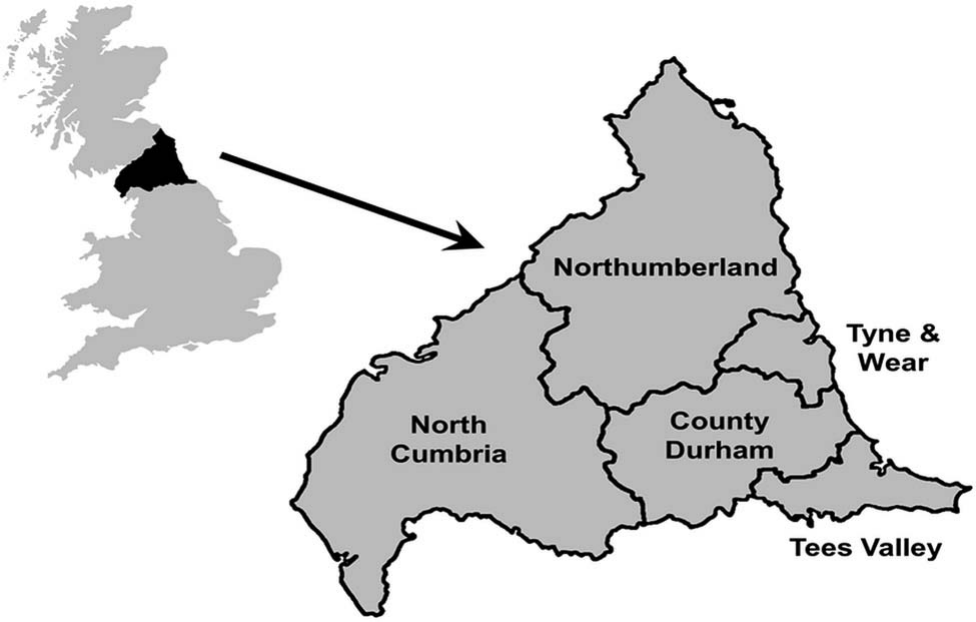
Map showing the region covered by the Northern Congenital Abnormality Survey (NorCAS) and the Northern Survey of Twin and Multiple Pregnancies (NorSTAMP).

The Northern Survey of Twin and Multiple Pregnancies (NorSTAMP) collects data on all multiple pregnancies to mothers residing in the North of England (figure 1). The North of England is a geographically defined area with a population of almost three million (with little immigration or emigration) and approximately 32 000 births per year. Multiple pregnancies are ascertained from the prenatal dating scan, the 20-week anomaly scan, and at delivery.^10^ In addition to basic maternal and fetal characteristics, chorionicity is recorded by NorSTAMP. Data on chorionicity is collected throughout pregnancy but the final diagnosis of chorionicity for twins of the same sex is based on placental examination and histology.^10^ If there is no pathological examination of the placenta, the diagnosis is made based on the prenatal ultrasound determination.

The NorSTAMP records are linked to the Northern Congenital Abnormality Survey (NorCAS). The NorCAS collects data on cases with congenital anomalies delivered to women residing in the North of England. Cases occurring in late miscarriages (20–23 weeks gestation), termination of pregnancy for fetal anomaly (TOPFA; any gestation), stillbirths (≥24 weeks gestation) and live births are notified to NorCAS. Cases are notified from multiple sources including antenatal ultrasound, fetal medicine, cytogenetic laboratories, the regional cardiology centre, pathology and paediatric surgery, ensuring high case ascertainment. Up to eight congenital anomalies per case are recorded.

Cases are coded according to the International Classification of Diseases (ICD) V.10. The European Surveillance of Congenital Anomalies (EUROCAT, a network of 38 registers in 20 European countries) exclusion list for minor anomalies is employed.^11^

Data on the annual number of live and stillbirths to mothers residing in the North of England (combined and by maternal age) was provided by the Office for National Statistics. Data on the annual number of twin live and stillbirths were provided by the NorSTAMP. The annual numbers of singleton births were calculated by subtracting the annual number of multiple births from the annual number of all births. Maternal age data were missing for 248 (2.1%) twin pregnancies and these were excluded from the denominator for analysis of maternal age.

### Ethical approval

Parental consent is required for NorSTAMP. The NorCAS has approval from the Confidentiality Advisory Group of the Health Research Authority (PIAG 2-08(e)/2012), to hold data without consent and ethics committee approval (09/H0405/48) to undertake studies involving the data.

### Case definition

All cases with a final diagnosis of CHD (ICD 10: Q20-26) notified between 1 January 1998 and 31 December 2010 were included. Cases with a minor CHD only, such as patent ductus arteriosus (PDA) with a gestational age <37 weeks, were excluded.^11^ Cases known to occur with extra-cardiac anomalies (ie, congenital anomalies not of the cardiovascular system) are likely to have different aetiologies than cases with isolated CHD. For example, CHD occurring with chromosomal/genetic anomalies may result directly from chromosomal aneuploidy.^12^ These cases are likely to have different risk factors, such as increased maternal age.^13^ ^14^ Analysis was performed on cases of isolated CHD only, to investigate the purest possible association between CHD and plurality.

### Case coding

Twins were coded as MC or DC. Due to small case numbers, it was not possible to analyse the association between plurality and CHD according to CHD subtype. However, it was possible to analyse groups of CHD subtypes, which were classified according to severity. Based on the classification system outlined by Khoshnood *et al*,^15^ cases of CHD were categorised as severe, moderate and mild CHD. However, we also included double outlet RV, interrupted aortic arch and mitral valve anomalies. The groups of CHD subtypes are shown in table 1. Cases with multiple CHD subtypes were categorised according to the CHD in the highest severity group. Cases included in Q20-26 but not described in one of the severity categories (eg, PDA ≥37 weeks gestation) remained unclassified.

**Table 1:** Frequency of CHD subtypes and severity categories according to plurality and chorionicity

CHD subtype	Twins (any chorionicity) N (% of 154)	Dichorionic twins N (% of 96)	Monochorionic twins N (% of 47)	Singletons N (% of 2984)
Severe CHD	7 (4.6)	4 (4.2)	3 (6.4)	132 (4.4)
Single ventricle	2 (1.3)	1 (1.0)	1 (2.1)	15 (0.5)
Hypoplastic left heart	2 (1.3)	1 (1.0)	1 (2.1)	76 (2.6)
Hypoplastic right heart	3 (2.0)	2 (2.1)	1 (2.1)	41 (1.4)
Moderate CHD	31 (20.1)	25 (26.0)	5 (10.6)	712 (23.9)
Pulmonary valve atresia	5 (3.3)	5 (5.2)	0	32 (1.1)
Common arterial trunk	0	0	0	20 (0.7)
Atrioventricular septal defect	2 (1.3)	1 (1)	1 (2.1)	70 (2.4)
Aortic valve atresia/stenosis	4 (2.6)	3 (3.1)	1 (2.1)	100 (3.4)
Transposition of the great vessels	2 (1.3)	1 (1.0)	1 (2.1)	145 (4.9)
Tetralogy of Fallot	6 (3.9)	5 (5.2)	1 (2.1)	117 (3.9)
Total anomalous pulmonary venous return	2 (1.3)	2 (2.1)	0	34 (1.1)
Coarctation of aorta	10 (6.5)	8 (8.3)	1 (2.1)	132 (4.4)
Double outlet RV	0	0	0	18 (0.6)
Interrupted aortic arch	0	0	0	11 (0.4)
Mitral valve anomalies	0	0	0	33 (1.1)
Mild CHD	106 (68.8)	63 (65.6)	35 (74.4)	1967 (69.9)
Ventricular septal defect	69 (44.8)	39 (40.6)	25 (53.2)	1392 (46.7)
Atrial septal defect	18 (11.7)	12 (12.5)	4 (8.5)	339 (11.4)
Pulmonary valve stenosis	19 (12.3)	12 (12.5)	6 (12.8)	236 (7.9)
Other CHD	10 (6.5)	4 (4.2)	4 (8.5)	173 (5.8)
Patent ductus arteriosus (≥37 weeks)	4 (2.6)	1 (1.0)	2 (4.3)	58 (1.9)
Total	154 (100.0)	96 (100.0)	47 (100.0)	2984 (100.0)

CHD, congenital heart disease.

### Statistical analysis

Total birth prevalence was calculated as the number of cases (in live births, late miscarriages, stillbirths or TOPFAs) per 10 000 live and stillbirths (total births).

The unadjusted RR of CHD in twins compared with singletons was estimated using Poisson regression models with the number of cases of CHD as the outcome, log(total births) as the offset and plurality (singleton or twin) as an explanatory variable. Adjusted RRs were estimated by refitting the models to include year of delivery (continuous variable) and maternal age (<20, 20–29, 30–34 and ≥35 years). The interaction between year of delivery and plurality was investigated by refitting the model with a cross-product term. The unadjusted RRs of CHD associated with maternal age and year of delivery were also estimated using Poisson regression.

All statistical analyses were performed in Stata V.13; p<0.05 was considered statistically significant.

## Results

Between 1998 and 2010, there were 399 414 singleton pregnancies and 6101 twin pregnancies that resulted in (at least one) live or stillbirth in the North of England. This equated to 11 871 total births, given that only one twin was live or stillborn in 331 pregnancies. Of the twins, 4359 pregnancies (8605 births, 72.5%) were DC and 1170 pregnancies (2317 births, 19.5%) were MC, leaving 542 pregnancies (949 births, 8.0%) with unknown chorionicity. The proportion of twin pregnancies increased from 2.6% in 1998 to 2.9% in 2010, although this did not reach statistical significance (test for trend: p=0.07).

There were 4160 cases of CHD delivered between 1998 and 2010: 3965 singletons and 187 twins. Of the 187 twins with CHD, 114 (61.0%) were DC, 60 (32.1%) were MC and 13 (7.0%) had unknown chorionicity.

### Extra-cardiac anomalies

Of the singletons with CHD, 700 (17.7%) occurred with chromosomal/genetic anomalies and 281 (7.1%) with structural anomalies. Of the twins with CHD, 15 (8.0%) occurred with chromosomal/genetic anomalies and 18 (9.6%) with structural anomalies. Twins with CHD were at significantly decreased risk of chromosomal/genetic anomalies compared with singletons (RR=0.45, 95% CI 0.28 to 0.74; p<0.001). The risk of structural anomalies was not significantly different in twins compared with singletons (RR=1.22, 95% CI 0.77 to 1.91; p=0.40). Cases with extra-cardiac anomalies were excluded from further analysis, leaving 2984 singletons and 154 twins with isolated CHD.

### CHD subtypes, severity and concordance

Of the singletons with isolated CHD, 132 (4.4%) had severe CHD, 721 (23.9%) had moderate CHD, 1967 (65.9%) had mild CHD and 173 (5.8%) were of unclassified severity. Of the twins, 7 (4.5%) had severe CHD, 31 (20.1%) had moderate CHD, 106 (68.8%) had mild CHD and 10 (6.5%) were of unclassified severity. The distribution of CHD subtypes and severity categories according to chorionicity is shown in table 1.

There were eight sets of twins with concordant CHD (four with the same subtype), of which six were DC and two were MC.

### Birth prevalence

There were 2984 singletons with isolated CHD, a prevalence of 74.7 per 10 000 total births (table 2); 0.7% of singleton pregnancies were associated with CHD. There were 154 twins with CHD, a prevalence of 129.7 per 10 000 total births; in 2.5% of twin pregnancies, at least one twin was affected by isolated CHD. Of the 154 twins with CHD, 96 occurred in DC and 47 in MC pregnancies, giving prevalence rates of 111.6 and 202.8 per 10 000 total births, respectively. At least one twin was affected by isolated CHD in 2.2% of DC twin pregnancies and 4.0% of MC twin pregnancies. The prevalence of severe, moderate and mild CHD are shown in table 2 by chorionicity. At least one twin was affected by severe, moderate and mild CHD in 0.1%, 0.5% and 1.7% of twin pregnancies, respectively.

**Table 2:** Prevalence per 10 000 total birth (95% CI) of CHD in twins and singletons, according to CHD severity and chorionicity

CHD severity	Twins			Singletons
	Twins (any chorionicity)	Dichorionic twins	Monochorionic twins	
All CHD	129.7 (110.2 to 151.7)	111.6 (90.5 to 136.1)	202.8 (149.4 to 268.8)	74.7 (72.1 to 77.4)
Severe CHD	5.9 (2.4 to 12.2)	4.6 (1.3 to 11.9)	12.9 (2.7 to 37.8)	3.3 (2.8 to 3.9)
Moderate CHD	26.1 (17.8 to 37.0)	29.1 (18.8 to 42.9)	21.6 (7.0 to 50.3)	17.8 (16.5 to 19.2)
Mild CHD	89.3 (73.2 to 107.9)	73.2 (56.3 to 93.6)	151.1 (105.4 to 209.5)	49.2 (47.1 to 51.5)

CHD, congenital heart disease.

### Maternal age

Among singletons, there was no evidence that CHD was associated with maternal age (p=0.53). Among twins, the association between CHD and maternal age was of borderline significance (p=0.07), with mothers aged <20 years having an increased risk of a pregnancy associated with CHD than mothers aged 20–29 years (table 3). Among DC twins, there was no evidence of an association between maternal age and CHD (p=0.41) (table 3). Among MC twins, there was evidence of an association between maternal age and CHD (p=0.01), with mothers aged <20 years being at increased risk of a pregnancy associated with CHD compared with mothers aged 20–29 years (table 3).

**Table 3:** RR of CHD according to maternal age and year of delivery

Maternal age at delivery*	N, Unadjusted RR (95% CI)			
	Twins (any chorionicity)	Dichorionic twins	Monochorionic twins	Singletons*
<20	N=14 RR=1.93 (0.96 to 3.88)	N=5 RR=1.06 (0.33 to 3.44)	N=8 RR=3.37 (1.27 to 8.95)	N=290 RR=0.94 (0.83 to 1.07)
20–29	N=66 RR=1 (reference)	N=40 RR=1 (reference)	N=21 RR=1 (reference)	N=1491 RR=1 (reference)
30–34	N=40 RR=0.74 (0.50 to 1.10)	N=25 RR=0.76 (0.46 to 1.26)	N=11 RR=0.64 (0.31 to 1.33)	N=754 RR=1.04 (0.95 to 1.13)
≥35	N=34 RR=0.97 (0.64 to 1.47)	N=26 RR=1.22 (0.75 to 2.01)	N=7 RR=0.63 (0.27 to 1.48)	N=422 RR=1.03 (0.93 to 1.15)
Year of delivery	RR=1.00 (0.96 to 1.04)	RR=0.96 (0.91 to 1.02)	RR=1.08 (1.01 to 1.18)	RR=0.98 (0.97 to 0.99)

*Twenty-nine (0.7%) singletons had missing maternal age data and were excluded. Maternal age data were missing in 2.1% of twins without CHD so these were excluded from the denominator.

CHD, congenital heart disease; RR, relative risk.

### Trends

The risk of CHD among singletons decreased significantly by 2% per year (p<0.001) (table 3). There was no evidence of a trend in CHD prevalence over time in twins (any chorionicity) (p=0.95) or in DC twins (p=0.09). In MC twins, the risk of CHD increased significantly by 8% per year (p=0.04) (table 3).

### Risk of CHD in twins versus singletons

Twins were at 73% significantly increased risk of CHD compared with singletons (p<0.001) (table 4). There was a 78%, 46% and 81% increased risk of severe, moderate and mild CHD in twins (any chorionicity) compared with singletons (p=0.135, p=0.037 and p<0.001, respectively) (table 4), although this only reached statistical significance for moderate and mild CHD.

**Table 4:** RR of CHD in twins versus singletons, according to CHD severity and chorionicity

CHD severity	Twins (any chorionicity) RR (95% CI); p value		Dichorionic twins RR (95% CI); p value		Monochorionic twins RR (95% CI); p value	
	Unadjusted	Adjusted*	Unadjusted	Adjusted*	Unadjusted	Adjusted*
All CHD	1.73 (1.48 to 2.04); p<0.001	1.75 (1.48 to 2.06); p<0.001	1.49 (1.22 to 1.83); p<0.001	1.51 (1.24 to 1.86); p<0.001	2.72 (2.04 to 3.62); p<0.001	2.76 (2.07 to 3.69); p<0.001
Severe CHD	1.78 (0.83 to 3.82); p=0.14	1.82 (0.85 to 3.90); p=0.12	1.41 (0.52 to 3.80); p=0.50	1.39 (0.51 to 3.76); p=0.52	3.92 (1.25 to 12.30); p=0.02	3.90 (1.24 to 12.27); p=0.02
Moderate CHD	1.46 (1.02 to 2.10); p=0.04	1.54 (1.07 to 2.20); p=0.02	1.63 (1.09 to 2.43); p=0.02	1.67 (1.12 to 2.49); p=0.01	1.21 (0.50 to 2.92); p=0.67	1.24 (0.51 to 2.98); p=0.64
Mild CHD	1.81 (1.49 to 2.20); p<0.001	1.80 (1.47 to 2.20); p<0.001	1.49 (1.16 to 1.91); p=0.002	1.50 (1.17 to 1.13); p=0.001	3.07 (2.20 to 4.28); p<0.001	3.12 (2.23 to 4.36); p<0.001

*Adjusted for year of delivery and maternal age. Maternal age was missing in 29 (0.7%) singleton cases and so these cases were excluded. Maternal age data were missing in 2.1% of twins without CHD so these were excluded from the denominator.

CHD, congenital heart disease; RR, relative risk.

MC twins were at 82% significantly increased risk of CHD compared with DC twins (RR=1.82, 95% CI 1.29 to 2.57; p<0.001). Compared with singletons, DC twins were at 49% significantly increased risk of CHD (p<0.001) and MC twins were at 172% significantly increased risk of CHD (p<0.001) (table 4). DC twins were at 41%, 63% and 49% increased risk of severe, moderate and mild CHD, respectively (table 4), although this did not reach statistical significance for severe CHD (p=0.50, p=0.02 and p=0.002, respectively). MC twins were at 292% significantly increased risk of severe CHD (p=0.02) and 207% significantly increased risk of mild CHD (p<0.001). There was no significant effect among moderate CHD (p=0.64) (table 4).

Adjusting for year of delivery and maternal age had little impact on the RR of CHD in twins compared with singletons (table 4).

When considering all twins (any chorionicity) and DC twins, the interaction between year of delivery and plurality was not significant (p=0.45 and p=0.52, respectively). Among MC twins, there was a significant interaction between year of delivery and plurality (p=0.01), with the RR of CHD in MC twins compared with singletons increasing over the study period (interaction term: RR=1.11, 95% CI 1.02 to 1.20).

## Discussion

In this population-based study, we found a 73% increased risk of CHD in twins compared with singletons. MC twins were at 172% and DC twins were at 49% increased risk of CHD compared with singletons.

This is one of few studies to examine the RR of CHD in twins compared with singletons. The primary strength of this study is the use of population-based data derived from an established, high-quality, congenital anomaly register. Multiple sources notify the register of cases, ensuring high case ascertainment. Accurate diagnoses are achieved by the review of complex cases by paediatric pathologists and clinical geneticists and, where relevant, diagnoses are confirmed via postmortem. By linking to a population-based register of multiple pregnancies, we were able to estimate the RR of CHD according to chorionicity, which few studies have accomplished.^1^ ^9^ Data on chorionicity is unlikely to be misclassified, given that the final diagnosis of like-sex twins is based on placental examination and histology.

A further strength is that CHD occurring in TOPFAs, late miscarriages and stillbirths were included. TOPFAs are less frequent in twin compared with singleton pregnancies, so had they been excluded; our RR of CHD associated with twins may have been overestimated.^16^ Stillbirth is more common in twin compared with singleton pregnancies, so excluding stillbirths could have diluted the RR of CHD.^16^

We examined the RR of CHD in twins versus singletons adjusted for confounding factors. Year of delivery is a potential confounder given that the twinning rate increased slightly over the study period. Maternal age may have been a confounder due to the association between increased maternal age and multiple pregnancy^17^ and the increased risk of CHD with increased maternal age, which is reported in some, but not all studies.^18–21^

This study has some limitations. First, the sample size was small meaning non-significant results could have resulted from type II errors. Among MC twins, the significant association with maternal age in under 20s should be interpreted cautiously due to low case numbers. Additionally, we were only able to examine severity categories as opposed to subtypes. As NorSTAMP requires parental consent, chorionicity data were not available for all twins. However, chorionicity data were missing for just 7% of cases and 8% of the denominator. Moreover, eight sets of twins with CHD were from the same pregnancy. This violates one of the assumptions of Poisson regression, that all observations should be independent. However, after excluding eight cases (one out of each set), the RR reduced only slightly (unadjusted RR=1.63, 95% CI 1.38 to 1.93; p<0.001, RR=1.40, 95% CI 1.14 to 1.73; p=0.002 and RR=2.60, 95% CI 1.94 to 3.49; p<0.001 for all twins (any chorionicity), DC twins and MC twins, respectively). We did not have data on zygosity as these are not recorded on the NorSTAMP. However, chorionicity can be used as a proxy zygosity given that all MC twins are monozygotic and most (∼90%) DC twins are dizygotic.^8^ Lastly, we were not able to investigate the risk associated with assisted reproductive technology (ART) as the registers are not able to hold this information.

Our 73% significant increased risk of CHD in twins compared with singletons is slightly greater than previously reported.^1^ ^4^ ^9^ Mastroiacovo *et al*^4^ reported an increased risk of 51% in Europe and Latin America (1978–1995), Glinianaia *et al*^1^ reported an increased risk of 47% in the North of England (using a subset of the present data, 1998–2002) and Herskind *et al*^9^ reported an increased risk of 63% in Denmark (1977–2001). In our study, the RR of CHD in MC twins increased over the study period, so we may have found a greater RR due to our more recent study period. The increase in risk may be a result of increased screening of MC twins, given that the increased risk of congenital anomaly in MC twins has become more widely known over time. In the UK, the National Institute for Health and Care Excellence (NICE)^22^ guidelines were updated in 2011 to recommend at least nine antenatal scans for MC twin pregnancy. This may have had particular impact if diagnosis of mild CHD improved, due to technical developments.

We identified a greater risk of CHD in MC compared with DC twins. Conversely, in the study by Glinianaia *et al*,^1^ there was no significant difference in the RR by chorionicity, but just nine cases in MC twins were examined. Herskind *et al*^9^ estimated the RR of CHD according to zygosity, finding no significant difference in risk. However, bias may have been incurred due to missing zygosity information. Indeed, in their cases with missing zygosity, the RR of CHD was greater than that of all twins (RR=2.41, 95% CI 2.07 to 2.80). Had a higher proportion of monozygotic twins had missing zygosity, this could partly explain why monozygotic twins were not at increased risk. Lastly, Herskind *et al*^9^ included only live births, which may have impacted on their results.

We found a significant increased risk of moderate and mild CHD in twins (any chorionicity) compared with singletons. While the risk of severe CHD was increased, it did not reach statistical significance, likely due to low power. The RR was significant among MC twins, due to the larger effect size, although this should be interpreted cautiously due to low sample size. Several studies have examined the RR of CHD in multiples compared with singletons by CHD subtype.^3^ ^4^ ^9^ Significant increased risks have been reported for ventricular septal defect (VSD), atrial septal defect, single ventricle, tetralogy of Fallot, atrioventricular septal defect and coarctation of aorta, although the effect sizes vary by study. Herskind *et al* uniquely examined subtypes according to zygosity, but could only examine VSD in monozygotic twins due to low sample size, finding a 73% increased risk compared with singletons.

The aetiology of CHD is becoming more researched and is hypothesised to be of both genetic and haemodynamic origin.^23^ The aetiology of the increased risk of CHD in twins is unresolved. Twin to twin transfusion in MC twins was identified as an important risk factor for CHD.^8^ ^24^ However, this does not explain why there would be an increased risk in DC twins. Others hypothesise that placental vascular anastomoses between the monozygotic co-twins’ circulations may lead to fluctuations in blood flow during fetal heart development, causing CHD.^25^ ^26^ If the aetiology of CHD in twins is predominantly haemodynamic as opposed to genetic, this may explain why chromosomal anomalies were less common in twins with CHD compared with singletons. Alternatively, monozygotic twinning itself is hypothesised to be part of a morphogenic anomaly which leads to a congenital anomaly.^27^ Given that all MC twins are monozygotic and around 10% of DC twins are monozygotic, this might explain why there was an increased risk in both MC and DC twins and why the effect size was greater in MC twins. However, previous research also found an increased risk among dizygotic twins.^9^ Perhaps the increased risk in DC twins could be related to the use of ART, which can result in twin pregnancy and has been linked to an increased CHD prevalence.^28^

**Key messages**

**What is already known on this subject?**

- Twins, in particular monochorionic twins, are at increased risk of congenital anomaly compared with singletons.
- Existing research suggests there is an increased risk of congenital heart disease (CHD) in twins compared with singletons.
- The effect of chorionicity and CHD severity on the increased risk in twins is less well researched.

**What might this study add?**

- Twins are at 73% increased risk of CHD compared with singletons.
- The risk among monochorionic (MC) twins exceeded that of dichorionic twins, with an increased risk of 82%.
- The prevalence of CHD in MC twins has increased over time.

**How might this impact on clinical practice?**

- Twin pregnancies, in particular MC twin pregnancies, require increased antenatal surveillance for CHD.
- This information is important for health professionals when counselling women with a twin pregnancy.
